# Chromosome-Scale Assembly and Annotation of Eight *Arabidopsis thaliana* Ecotypes

**DOI:** 10.1093/gbe/evae169

**Published:** 2024-08-05

**Authors:** Zachary Kileeg, Pauline Wang, G Adam Mott

**Affiliations:** Department of Biological Sciences, University of Toronto—Scarborough, Toronto, Canada; Department of Cell and Systems Biology, University of Toronto, Toronto, Canada; Department of Cell and Systems Biology, University of Toronto, Toronto, Canada; Centre for the Analysis of Genome Evolution & Function, University of Toronto, Toronto, Canada; Department of Biological Sciences, University of Toronto—Scarborough, Toronto, Canada; Department of Cell and Systems Biology, University of Toronto, Toronto, Canada; Centre for the Analysis of Genome Evolution & Function, University of Toronto, Toronto, Canada

**Keywords:** *Arabidopsis*, ecotypes, natural variation, long-read sequencing, structural variation, pan-genome

## Abstract

The plant *Arabidopsis thaliana* is a model system used by researchers through much of plant research. Recent efforts have focused on discovering the genomic variation found in naturally occurring ecotypes isolated from around the world. These ecotypes have come from diverse climates and therefore have faced and adapted to a variety of abiotic and biotic stressors. The sequencing and comparative analysis of these genomes can offer insight into the adaptive strategies of plants. While there are a large number of ecotype genome sequences available, the majority were created using short-read technology. Mapping of short-reads containing structural variation to a reference genome bereft of that variation leads to incorrect mapping of those reads, resulting in a loss of genetic information and introduction of false heterozygosity. For this reason, long-read de novo sequencing of genomes is required to resolve structural variation events. In this article, we sequenced the genomes of eight natural variants of *A. thaliana* using nanopore sequencing. This resulted in highly contiguous assemblies with >95% of the genome contained within five contigs. The sequencing results from this study include five ecotypes from relict and African populations, an area of untapped genetic diversity. With this study, we increase the knowledge of diversity we have across *A. thaliana* ecotypes and contribute to ongoing production of an *A. thaliana* pan-genome.

**Significance**

The plant *Arabidopsis thaliana* is used as a model organism for many crop studies. Increasing use of long-read sequencing has revealed the extent of existing structural variation is much greater than originally believed. As structural variation is often associated with different agronomic traits, identification and understanding of these regions may show new novel features and regions to analyze for novel functions. We present eight new sequenced ecotypes of *A. thaliana,* including five from relict and African populations, both of which are under-sampled. This study increases our knowledge of structural and genetic diversity within *A. thaliana* and contributes to plant research as a whole.

## Introduction

*Arabidopsis thaliana* is a model organism used as the basis for studies of many aspects of plant biology. *A. thaliana* is part of the brassicales family, which includes a variety of oilseed crops of agronomic importance (Raza et al. 2020). *A. thaliana* is a predominantly self-fertilizing plant, with an estimated 3% out-crossing (Platt et al. 2010). In addition to its roles as a general plant model system and a tool for the study of brassicas, due to the depth of genomic resources available *A. thaliana* is also used as a reference for genome assembly and comparison (Lijavetzky et al. 2003; Long et al. 2007; Provart et al. 2016). *A. thaliana* is an excellent genomic system as it is diploid and has a relatively small ∼135 mb genome (Hays 2002; Hou et al. 2022). This makes it very amenable to sequencing, genetic analysis, and computational benchmarking (Hays 2002; Hou et al. 2022).

Naturally occurring variants of *A. thaliana*, also known as ecotypes, have been isolated from many regions including Europe, Africa, North America, Japan, and South Korea (Koornneef et al. 2004; The 1001 Genomes Consortium 2016). The diverse environmental conditions of these geographic regions have led to natural genetic variation across these ecotypes, providing insights into plant adaptation to abiotic and biotic stress (Coolen et al. 2019). Sequencing efforts by the 1001 genomes consortium have identified extensive heterozygosity and polymorphisms across *A. thaliana* world-wide, highlighting the natural variation inherent to this plant species (The 1001 Genomes Consortium 2016). Recent work has brought into question how much of this diversity has been properly captured. Performing de novo long-read sequencing of several ecotypes isolated from Sweden found that a significant portion of the heterozygosity observed was the result of spurious single-nucleotide polymorphisms caused by the use of short-read sequencing (Jaegle et al. 2023). In place of the heterozygosity, the long-read sequencing identified significant levels of structural variation that could not be identified using short-read sequencing (Jaegle et al. 2023). This work highlights that the mismapping of reads derived from loci of structural variation in new ecotypes will simultaneously lead to a loss of genetic information and the introduction of false heterozygosity (Jaegle et al. 2023). Long-read sequencing and de novo genome assembly is the only way to properly identify structural variation and novel genetic information.

With the increasing availability of long-read sequencing, many recent studies have performed resequencing of a variety of *A. thaliana* ecotypes to capture the structural variation and natural diversity of the species (Jiao and Schneeberger 2020; Kang et al. 2023; Wlodzimierz et al. 2023; Lian et al. 2024). These studies have indicated that Eurasian ecotypes are over-sampled, with the discovery of new genetic diversity reaching saturation. In contrast, the same studies found that African and relict populations are under-sampled and that the discovery of new genetic diversity has not yet reached saturation in these populations. Consistent with this, genetic analysis of *A. thaliana* ecotypes also showed greater genetic divergence across African and relict populations than Eurasian populations (Durvasula et al. 2017). It is therefore clear that additional long-read sequencing of relict and African populations is likely to uncover novel genetic diversity in *A. thaliana*.

To this end, we sequenced the genomes of eight different ecotypes of *A. thaliana* from diverse geographic regions, including two African and three Eurasian relict ecotypes. The resulting genomes are of high quality and continuity, comparable to the TAIR10.1 reference. These results expand the overall cache of long-read sequenced ecotype genomes available to researchers, offering additional resources for the discovery of novel genetic variation and improving comparative analyses across *A. thaliana.*

## Results and Discussion

### Assembly and Gene Prediction Assessment

**Fig. 1.**
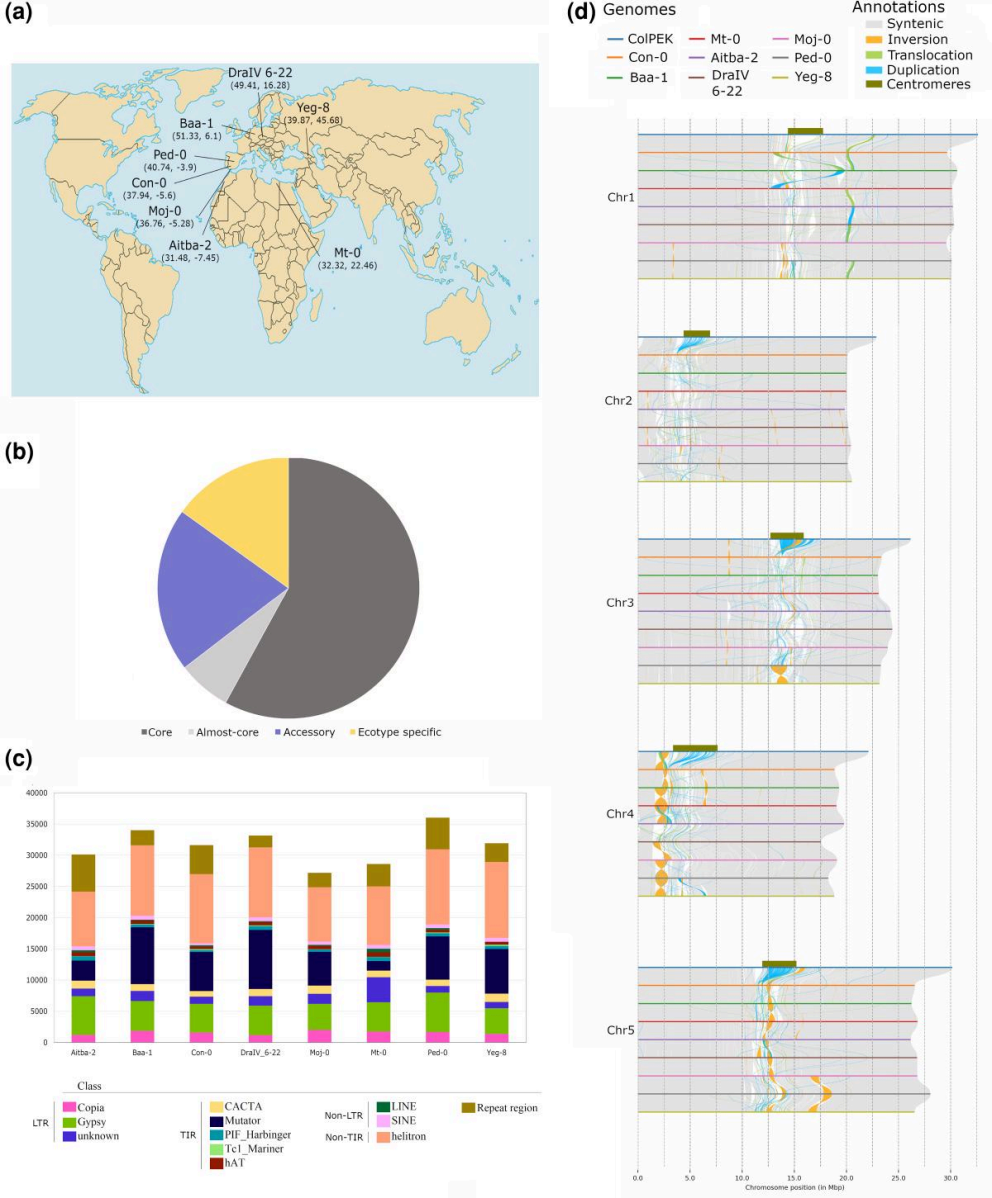
Analysis of eight *A. thaliana* ecotype genomes. a) Location of isolation shown for chosen ecotypes labeled with coordinates listed in parentheses. b) Orthogroup assignment for predicted protein-coding genes across the eight ecotypes are labeled for core (dark gray; all eight ecotypes contain an orthologue), almost core (light gray; seven ecotypes contain an orthologue), accessory (purple; two to six ecotypes contain an orthologue), and ecotype specific (yellow; one ecotype contains a given gene). c) De novo transposon identification. Each ecotype is a single bar; each type of repeat element is shown in a unique color. The total number of predicted transposable elements of each class is shown under each bar. d) Synteny analysis for the five main chromosomes across the eight *A. thaliana* ecotypes compared to ColPEK. Major structural rearrangements are denoted by colored lines connecting involved chromosomes. Orange = inversion event, green = translocation event, and blue = duplication event. Dark green bars represent the location of the centromere. Map adapted from Wikimedia Commons user STyx.

The eight *Arabidopsis* ecotypes were selected from a collection of Eurasian, Eurasian relict, and African accessions (Fig. 1a). Ecotype genomes were sequenced to a coverage of 16 to 100× with long nanopore reads and polished using short Illumina reads with a coverage >40×. Sequences were assembled into five pseudo-chromosomes covering 119–121 Mb (Table 1, Fig. 1). This is a slightly shorter total length compared to the *A. thaliana* ColPEK and ColCEN references, with sizes of ∼135 Mb (Naish et al. 2021; Hou et al. 2022), but is comparable to the TAIR10.1 assembly (Lamesch et al. 2012). Chromosome lengths of the five main chromosomes were all similar to the TAIR10.1 reference (Table 1). Genomes contained high contiguity, with contig N50s > 8.9 Mb, scaffold N50s > 23 Mb, and L50s of 2–4 (Table 1). These benchmarks are also slightly lower than ColCEN and ColPEK, but comparable to the TAIR10.1 assembly and other recent genome assemblies (Jiao and Schneeberger 2020). All genomes contain a GC% of ∼36%, consistent with the TAIR10.1 reference (Table 1). On average, over 88% of Illumina short-reads mapped to the draft genomes during the polishing steps (Table 1).

**Table 1:** Genome assembly and annotation quality for 8 *A. thaliana* ecotypes

	Aitba-2	Baa-1	Con-0	DraIV 6-22	Moj-0	Mt-0	Ped-0	Yeg-8
Genome assembly								
Genome size (Mb)	121.47	121.26	128.34	121.33	121.19	120.89	122.76	121.32
Contigs/scaffolds	355/170	230/101	2,838/1,260	155/80	176/58	272/114	333/150	231/71
Contig N50 (Mb)	12.42	8.92	11.00	11.06	19.07	13.60	11.69	15.87
Scaffold N50 (Mb)	24.27	23.90	23.36	24.34	23.92	23.05	23.29	23.11
L50	4	4	2	4	2	4	4	2
# of reads	845,646	347,717	3,987,223	290,415	508,902	626,510	404,542	635,837
# of bases (Mb) in reads	3961.14	2156.02	13831.80	1968.44	3338.90	3023.10	2236.27	3531.79
Approximate coverage (X)	32.61	17.78	107.78	16.22	27.55	25.01	18.22	29.11
Average % mapping short-reads for polishing	88.78	88.16	94.75	96.08	93.72	96.76	95.85	92.52
K-mer completeness	94.80	97.91	97.30	93.68	98.76	89.35	95.59	96.35
QV (consensus.30 = 99.9% probability)	39.86	34.57	36.77	31.17	31.96	24.52	28.97	38.57
% GC content	36.11	36.11	36.57	36.09	36.12	36.12	36.11	36.06
% BUSCO completeness (single, duplicated):genome	99.20 (S:98.3, D:0.9)	99.30 (S:98.5, D:0.8)	99.40 (S:98.5, D:0.9)	99.10 (S:98.3, D:0.8)	99.10 (S:98.3, D:0.8)	99.20 (S:98.3, D:0.9)	99.10 (S:98.3, D:0.8)	99.30 (S:98.4, D:0.9)
Chromosome lengths (Mb)								
Chr1	30.26	30.63	29.69	30.32	29.60	30.15	30.38	30.08
Chr2	19.87	20.02	20.75	20.16	20.43	20.03	20.22	20.51
Chr3	24.27	23.02	23.55	24.41	23.97	23.11	23.38	23.17
Chr4	19.78	19.28	19.40	17.56	19.07	19.04	18.32	18.82
Chr5	26.17	26.25	26.79	26.80	26.80	26.83	28.11	26.54
Gene annotation								
Protein-coding genes (liftoff)	26,499	26,666	26,473	26,671	26,423	26,738	26,323	26,598
Protein-coding genes (de novo*, unfiltered)*	32,744	32,882	35,979	32,930	33,353	33,157	33,303	32,698
% BUSCO completeness (single, duplicated):proteins	99.1 (S:97.8, D:1.3)	99.1 (S:97.8, D:1.3)	98.9 (S:97.6, D:1.3)	98.9 (S:97.7, D:1.2)	98.7 (S:97.5, D:1.2)	98.8 (S:97.3, D:1.5)	99.1 (S:97.6, D:1.5)	99.1 (S:97.8, D:1.3)
% of Araport11 blast mapping (>50% similarity >50 bitscore)	96.92	97.03	97.16	97.04	96.84	96.80	96.80	96.90
% of Araport11 liftoff mapping	95.82	96.42	95.73	96.44	95.55	96.68	95.18	96.18
% BP masked (TEs)	14.52	14.91	13.92	14.57	13.21	13.49	15.26	13.83

BUSCO and k-mer counting were used to assess genome completeness. BUSCO scores were comparable to TAIR10.1, showing completeness of 99% or greater for all genomes (Table 1). K-mer counting using Merqury showed genome completion >90% and QV >30 for all genomes except Mt-0, which showed a completeness of 89% and QV of 24, and Ped-0, which showed a QV of 29 (Table 1). Assembly quality was similar to that of the TAIR10.1 assembly and recent genome assemblies (Jiao and Schneeberger 2020).

Protein sequences were inferred using ab initio, homology, and transcript data. There were ∼32,000–36,000 protein-coding sequences predicted using the combined methods (Table 1). Using homology methods from liftoff, we also predicted over 26,000 protein-coding genes using the Araport11 reference. The large disparity most likely comes from the filtering methods used. Our protein predictions likely overestimate the true number of protein-coding genes, but are more amenable for downstream filtering and analysis. The liftoff predictions instead are only protein-coding genes with strong homology to the existing Araport11 reference set. Using homology-based inference methods (liftoff and blastp), the predicted protein sets showed 96% to 98% and 95% to 98% completeness, respectively. In agreement with this observation, we assessed the protein-set completeness with BUSCO and found completeness percentages of >98.7 across all ecotypes (Table 1). Taken together, these analyses indicate that a large portion of the protein-coding sequences have been captured.

### Orthogroup Prediction

The protein sequences were then used to construct orthogroups using Orthofinder. Across the eight ecotypes, the proteins clustered into ∼44,000 orthogroups (Fig. 1b). We found ∼64% of orthogroups were core or almost core (≥7 ecotypes contained an orthologue) across the ecotypes (Fig. 1b). In contrast, ∼20% were accessory genes (two to six ecotypes contained an orthologue), and 15% were ecotype specific (Fig. 1b). This distribution was similar to that previously found across 69 ecotypes (Lian et al. 2024).

### Transposable Element Analysis

Transposon elements (TEs) were predicted de novo using EDTA and AnnoSINE_v2. EDTA was used for annotations of most TEs, while AnnoSINE_v2 was used to predict SINEs that were not captured in the EDTA analysis. Repetitive elements indicative of TEs were predicted to comprise ∼13%–15% of the genomes (Table 1, Fig. 1, supplementary table S1, Supplementary Material online). Helitrons were the most common type, with TIR class being the second most abundant (Fig. 1c). SINE occurrence was similar across all ecotypes except for Con-0, which had much fewer (Fig. 1c). The occurrence of LINEs was variable across ecotypes, with Yeg-8 having the fewest and Mt-0 having the most (Fig. 1c).

### Synteny and Structural Variation

Syntenic relationships between each of the ecotypes and ColPEK were inferred using SYRI. Syntenic relationships showed that the chromosomes are largely conserved across the ecotypes (Fig. 1). The largest area of variation occurred across and around the centromeres, with increased duplication and inversion across the ecotypes (Fig. 1). Interestingly, it appears pericentromeric regions experienced the highest diversity across ecotypes (Fig. 1), though this finding may be confounded by a lack of quality assembly surrounding the centromeres in the ecotypes sequenced here. There also appear to be regions where structural variation is more likely. For example, the region around position 20 to 25 Mb in chromosome 1 appears to be a hot spot for duplication and translocation (Fig. 1). Interestingly, this region has been reported to contain a high number of biotic stress resistance genes (Van de Weyer et al. 2019). It may be that the observed structural variation is indicative of stress adaptation.

## Methods

### Plant Material and Growth Conditions

*Arabidopsis* seeds from ecotypes Aitba-2, Baa-1, Con-0, DraIV 6-22, Moj-0, Mt-0, Ped-0, and Yeg-8 were acquired from the Arabidopsis Biological Resource Center. Natural variant geographic location and stock information are outlined in supplementary table S2, Supplementary Material online. Seeds were vernalized at 4 °C for 1 to 4 weeks on 2.2 g/L Murashige and Skoog basal medium (Sigma). The plates were then transferred into controlled growth chambers at 22 °C under long-day light conditions (16 h light and 8 h dark). After 7 days, seedlings were transferred to soil supplemented with 1 g/L fertilizer with a ratio 20P:20N:20K (Plant-pro). Plants were watered as needed and every 2 weeks the watering was supplemented with 0.5 g/L additional fertilizer. After ∼3 weeks, plants were transferred to short-day conditions (12 h light and 12 h dark) for 1 week to reduce stress and starch levels. All growth chambers provided light intensities of 120 µmol/m^2^ s and relative humidity of 40% to 60%. At 48 h prior to DNA extraction, plants were transferred into 24-h dark conditions to reduce levels of contaminating polysaccharides and polyphenols.

### DNA Extraction and Quality Assessment

High molecular weight DNA was extracted using the Macherey–Nagel HMW Nucleobond isolation kit as per manufacturer's instructions. DNA purity was estimated by measuring OD A260/280 and A260/230 ratios using a nanodrop spectrophotometer (Nanodrop 2000, Thermo-Fischer). DNA quality and fragmentation were visualized by gel electrophoresis.

### Library Construction and Genome Sequencing

Genomic DNAs were quantified with the Qubit instrument using the dsDNA BR Assay Kit (Thermo Fisher Scientific, Waltham, MA, USA). Approximately 2 mg of each gDNA was used to prepare sequencing libraries with the SQK-LSK109 Ligation Sequencing Kit and the EXP-NBD104 Native Barcoding Expansion Kit (Nanopore, Oxford, UK). Libraries were prepared and barcoded following the manufacturer's instructions. The barcoded libraries were pooled equally and sequenced on a PromethION R9.4.1 flow cell. Sequencing was performed for 68 h.

### Genome Assembly and QC

Computations were performed on Alliance Canada (formerly SciNet) computing resources (Loken et al. 2010). Following sequencing, adaptors were removed, and the raw reads were trimmed and corrected using porechop v0.2.4 (https://github.com/rrwick/Porechop) and filtlong v0.2.1 (https://github.com/rrwick/Filtlong). Final genome sizes were estimated using jellyfish v2.3.0 (Vurture et al. 2017) and Genomescope v2.0 (Ranallo-Benavidez et al. 2020) using default settings. For initial genome assembly, raw reads were assembled into contigs using flye v2.9.2 (Kolmogorov et al. 2019) with settings “–nano-raw –polish-target –threads 32” as flye uses its own correction. Draft assemblies were polished three times by aligning short-reads (source in supplementary table S3, Supplementary Material online) to the draft genomes using Bowtie2 v2.4.4 (Langmead and Salzberg 2012), then polishing with Pilon v1.24 (Walker et al. 2014). The average number of short-reads mapping to the target genome across all three iterations is found in Table 1. First-round polished assemblies were then scaffolded using RagTag v2.1.0 (Alonge et al. 2019) against the ColCEN (Naish et al. 2021) and ColPEK (Hou et al. 2022) telomere-to-telomere assembly to produce pseudo-chromosome level assemblies. As ColPEK does not contain assembled chloroplast or mitochondrial chromosomes, assemblies were first scaffolded against ColCEN, followed by scaffolding against ColPEK. Where contigs could be resolved into chloroplast or mitochondrial chromosomes, they were scaffolded. If they could not be resolved, they were left as separate contigs, and chloroplast/mitochondrial chromosomes were not labeled. Assemblies were then corrected three times by aligning the trimmed and filtered long-reads to the scaffolded genomes using minimap2 v2.24 (Li 2018, 2021) and then by polishing using racon v1.4.13 (Vaser et al. 2017). Assemblies then underwent another three rounds of polishing with short-reads by Bowtie2 and pilon.

For quality control, contigs were aligned to ColCEN using blastn v2.12.0 (Altschul et al. 1990) and removed if there was no significant match (*E*-value < 0.05). Genome continuity and completeness were measured by BUSCO v5.5.0 (Manni et al. 2021) using the embryophyte-odb10 set under mode “genome.” K-mer completeness and QV were measured using Merqury v1.3 (Rhie et al. 2020). Basic genome assembly statistics were found using assembly-stats v1.01 (https://github.com/sanger-pathogens/assembly-stats).

### Gene Prediction

Gene models were predicted using a combination of de novo, transcript, and protein inference data following the protocol of Lian et al. (2024). In short, de novo gene prediction was performed using Augustus (Stanke et al. 2006; Ter-Hovhannisyan et al. 2008), glimmerHMM v3.0.4 (Majoros et al. 2004), and the GeMoMa pipeline v1.9 (Keilwagen et al. 2019). 243 paired-end and single-end RNA sequencing read sets from 20 ecotypes were downloaded from NCBI SRA and used to infer transcripts (supplementary table S4, Supplementary Material online). Adapter presence and read quality were determined using fastqQCv0.11.9 (https://github.com/s-andrews/FastQC). Reads were trimmed as necessary using trimmomatic v0.39 (Bolger et al. 2014). Reads from RNA-seq datasets were aligned to each ecotype genome using Hisat2 v2.2.1 (Kim et al. 2019), and transcripts were assembled using Stringtie v2.2.1 (Pertea et al. 2015) and reconstructed using TransDecoder v5.7.1 (Haas, BJ. https://github.com/TransDecoder/TransDecoder). Lastly, protein sequences annotated from Araport11 (Cheng et al. 2017) were aligned to each ecotype genome using exonerate v2.4.0 (Slater and Birney 2005) using the protein2genome mode and from liftoff v1.6.3 (Shumate and Salzberg 2021) with settings “-p 8 -copies -sc 0.90 -exclude_partial -a 0.9 –polish.” All modes of evidence were combined using EVidenceModeler v2.1.0 (Haas et al. 2008). Gene prediction annotations were merged, and the longest isoform predicted and extracted as the representative sequence using AGAT v1.3.3 (https://github.com/NBISweden/AGAT).

To estimate protein-coding set completeness, homology was tested using liftoff and blastp v2.12.0 (Camacho et al. 2009) compared to the Araport11 annotations. Proteins were considered homologous if blastp bit scores were ≥50, and sequence similarity was ≥50% following the approach of Pearson (Pearson 2013). Protein-set completeness was also measured with BUSCO v5.5.0 using the embryophyte-odb10 set under mode “proteins.”

### Gene Orthology Analysis

Orthogroups were determined using Orthofinder v2.5.5 (Emms and Kelly 2019). Orthogroups were split into core (all eight ecotypes contain an orthologous gene), almost core (only seven ecotypes contain an orthologous gene), accessory (two to six ecotypes contain an orthologous gene), and ecotype specific (gene found in a single ecotype).

### Synteny Analysis

Whole genome alignments were performed using nucmer v3.1 (Kurtz et al. 2004), and syntenic analysis of the genomes was carried out using SYRI v1.6.3 (Goel et al. 2019). The five main chromosomes were compared sequentially to one another, starting with ColPEK, followed by Con-0, and continued until all ecotypes were added. Synteny maps were plotted using Plotsr v1.1.0 (Goel and Schneeberger 2022).

### Transposon Analysis

Repetitive elements were inferred de novo using the Extensive de novo TE Annotator v2.2 (Ou et al. 2019) with default settings. Transposon class was inferred for LTRs, TIRs, non-LTRs, non-TIRs, and repeat regions. For SINE inference, AnnoSINE v2 (Li et al. 2022) was used with default settings.

## Supplementary Material

evae169_Supplementary_Data

## Data Availability

Raw reads with adapters removed and genome assemblies with basic gene annotation are available at the European Nucleotide Archive under project PRJEB74780. Reads are deposited under accessions ERR12915332, ERR12912245, ERR12911107, ERR12911013, ERR12911012, ERR12910699, ERR12910384, and ERR12906128. Genome assemblies with annotations are deposited under accessions GCA_964036055.1, GCA_964036065.1, GCA_964036075.1, GCA_964036085.1, GCA_964036095.1, GCA_964036105.1, GCA_964036115.1, and GCA_964036125.1.

## References

[evae169-B1] Alonge M, Soyk S, Ramakrishnan S, Wang X, Goodwin S, Sedlazeck FJ, Lippman ZB, Schatz MC. RaGOO: fast and accurate reference-guided scaffolding of draft genomes. Genome Biol. 2019:20(1):224. 10.1186/s13059-019-1829-6. 31661016 PMC6816165

[evae169-B2] Altschul SF, Gish W, Miller W, Myers EW, Lipman DJ. Basic local alignment search tool. J Mol Biol. 1990:215(3):403–410. 10.1016/S0022-2836(05)80360-2. 2231712

[evae169-B3] Bolger AM, Lohse M, Usadel B. Trimmomatic: a flexible trimmer for Illumina sequence data. Bioinformatics. 2014:30(15):2114–2120. 10.1093/bioinformatics/btu170. 24695404 PMC4103590

[evae169-B4] Camacho C, Coulouris G, Avagyan V, Ma N, Papadopoulos J, Bealer K, Madden TL. BLAST+: architecture and applications. BMC Bioinformatics. 2009:10:421. 10.1186/1471-2105-10-421. 20003500 PMC2803857

[evae169-B5] Cheng C-Y, Krishnakumar V, Chan AP, Thibaud-Nissen F, Schobel S, Town CD. Araport11: a complete reannotation of the *Arabidopsis thaliana* reference genome. Plant J. 2017:89(4):789–804. 10.1111/tpj.13415. 27862469

[evae169-B6] Coolen S, Van Pelt JA, Van Wees SCM, Pieterse CMJ. Mining the natural genetic variation in *Arabidopsis thaliana* for adaptation to sequential abiotic and biotic stresses. Planta. 2019:249(4):1087–1105. 10.1007/s00425-018-3065-9. 30547240

[evae169-B7] Durvasula A, Fulgione A, Gutaker RM, Alacakaptan SI, Flood PJ, Neto C, Tsuchimatsu T, Burbano HA, Picó FX, Alonso-Blanco C, et al African genomes illuminate the early history and transition to selfing in *Arabidopsis thaliana*. Proc Natl Acad Sci U S A. 2017:114(20):5213–5218. 10.1073/pnas.1616736114. 28473417 PMC5441814

[evae169-B8] Emms DM, Kelly S. OrthoFinder: phylogenetic orthology inference for comparative genomics. Genome Biol. 2019:20(1):238. 10.1186/s13059-019-1832-y. 31727128 PMC6857279

[evae169-B9] Goel M, Schneeberger K. Plotsr: visualizing structural similarities and rearrangements between multiple genomes. Bioinformatics. 2022:38(10):2922–2926. 10.1093/bioinformatics/btac196. 35561173 PMC9113368

[evae169-B10] Goel M, Sun H, Jiao W-B, Schneeberger K. SyRI: finding genomic rearrangements and local sequence differences from whole-genome assemblies. Genome Biol. 2019:20(1):277. 10.1186/s13059-019-1911-0. 31842948 PMC6913012

[evae169-B11] Haas BJ, Salzberg SL, Zhu W, Pertea M, Allen JE, Orvis J, White O, Buell CR, Wortman JR. Automated eukaryotic gene structure annotation using EVidenceModeler and the Program to Assemble Spliced Alignments. Genome Biol. 2008:9(1):R7. 10.1186/gb-2008-9-1-r7. 18190707 PMC2395244

[evae169-B12] Hays JB . *Arabidopsis thaliana*, a versatile model system for study of eukaryotic genome-maintenance functions. DNA Repair (Amst). 2002:1(8):579–600. 10.1016/S1568-7864(02)00093-9. 12509283

[evae169-B13] Hou X, Wang D, Cheng Z, Wang Y, Jiao Y. A near-complete assembly of an *Arabidopsis thaliana* genome. Mol Plant. 2022:15(8):1247–1250. 10.1016/j.molp.2022.05.014. 35655433

[evae169-B14] Jaegle B, Pisupati R, Soto-Jiménez LM, Burns R, Rabanal FA, Nordborg M. Extensive sequence duplication in *Arabidopsis* revealed by pseudo-heterozygosity. Genome Biol. 2023:24(1):44. 10.1186/s13059-023-02875-3. 36895055 PMC9999624

[evae169-B15] Jiao W-B, Schneeberger K. Chromosome-level assemblies of multiple *Arabidopsis* genomes reveal hotspots of rearrangements with altered evolutionary dynamics. Nat Commun. 2020:11(1):989. 10.1038/s41467-020-14779-y. 32080174 PMC7033125

[evae169-B16] Kang M, Wu H, Liu H, Liu W, Zhu M, Han Y, Liu W, Chen C, Song Y, Tan L, et al The pan-genome and local adaptation of *Arabidopsis thaliana*. Nat Commun. 2023:14(1):6259. 10.1038/s41467-023-42029-4. 37802986 PMC10558531

[evae169-B17] Keilwagen J, Hartung F, Grau J. Gemoma: homology-based gene prediction utilizing intron position conservation and RNA-seq data. Methods Mol Biol. 2019:1962:161–177. 10.1007/978-1-4939-9173-0_9. 31020559

[evae169-B18] Kim D, Paggi JM, Park C, Bennett C, Salzberg SL. Graph-based genome alignment and genotyping with HISAT2 and HISAT-genotype. Nat Biotechnol. 2019:37(8):907–915. 10.1038/s41587-019-0201-4. 31375807 PMC7605509

[evae169-B19] Kolmogorov M, Yuan J, Lin Y, Pevzner PA. Assembly of long, error-prone reads using repeat graphs. Nat Biotechnol. 2019:37(5):540–546. 10.1038/s41587-019-0072-8. 30936562

[evae169-B20] Koornneef M, Alonso-Blanco C, Vreugdenhil D. Naturally occurring genetic variation in *Arabidopsis thaliana*. Annu Rev Plant Biol. 2004:55(1):141–172. 10.1146/annurev.arplant.55.031903.141605. 15377217

[evae169-B21] Kurtz S, Phillippy A, Delcher AL, Smoot M, Shumway M, Antonescu C, Salzberg SL. Versatile and open software for comparing large genomes. Genome Biol. 2004:5(2):R12. 10.1186/gb-2004-5-2-r12. 14759262 PMC395750

[evae169-B22] Lamesch P, Berardini TZ, Li D, Swarbreck D, Wilks C, Sasidharan R, Muller R, Dreher K, Alexander DL, Garcia-Hernandez M, et al The Arabidopsis Information Resource (TAIR): improved gene annotation and new tools. Nucleic Acids Res. 2012:40(D1):D1202–D1210. 10.1093/nar/gkr1090. 22140109 PMC3245047

[evae169-B23] Langmead B, Salzberg SL. Fast gapped-read alignment with Bowtie 2. Nat Methods. 2012:9(4):357–359. 10.1038/nmeth.1923. 22388286 PMC3322381

[evae169-B24] Li H . Minimap2: pairwise alignment for nucleotide sequences. Bioinformatics. 2018:34(18):3094–3100. 10.1093/bioinformatics/bty191. 29750242 PMC6137996

[evae169-B25] Li H . New strategies to improve minimap2 alignment accuracy. Bioinformatics. 2021:37(23):4572–4574. 10.1093/bioinformatics/btab705. 34623391 PMC8652018

[evae169-B26] Li Y, Jiang N, Sun Y. AnnoSINE : a short interspersed nuclear elements annotation tool for plant genomes. Plant Physiol. 2022:188(2):955–970. 10.1093/plphys/kiab524. 34792587 PMC8825457

[evae169-B27] Lian Q, Huettel B, Walkemeier B, Mayjonade B, Lopez-Roques C, Gil L, Roux F, Schneeberger K, Mercier R. A pan-genome of 69 *Arabidopsis thaliana* accessions reveals a conserved genome structure throughout the global species range. Nat Genet. 2024:56(5):982–991. 10.1038/s41588-024-01715-9. 38605175 PMC11096106

[evae169-B28] Lijavetzky D, Carbonero P, Vicente-Carbajosa J. Genome-wide comparative phylogenetic analysis of the rice and Arabidopsis Dof gene families. BMC Evol Biol. 2003:3:17. 10.1186/1471-2148-3-17. 12877745 PMC184357

[evae169-B29] Loken C, Gruner D, Groer L, Peltier R, Bunn N, Craig M, Henriques T, Dempsey J, Yu C-H, Chen J, et al SciNet: lessons learned from building a power-efficient top-20 system and data centre. J Phys Conf Ser. 2010:256:012026. 10.1088/1742-6596/256/1/012026.

[evae169-B30] Long Y, Shi J, Qiu D, Li R, Zhang C, Wang J, Hou J, Zhao J, Shi L, Park B-S, et al Flowering time quantitative trait loci analysis of oilseed brassica in multiple environments and genomewide alignment with Arabidopsis. Genetics. 2007:177(4):2433–2444. 10.1534/genetics.107.080705. 18073439 PMC2219480

[evae169-B31] Majoros WH, Pertea M, Salzberg SL. TigrScan and GlimmerHMM: two open source ab initio eukaryotic gene-finders. Bioinformatics. 2004:20(16):2878–2879. 10.1093/bioinformatics/bth315. 15145805

[evae169-B32] Manni M, Berkeley MR, Seppey M, Simão FA, Zdobnov EM. BUSCO update: novel and streamlined workflows along with broader and deeper phylogenetic coverage for scoring of eukaryotic, prokaryotic, and viral genomes. Mol Biol Evol. 2021:38(10):4647–4654. 10.1093/molbev/msab199. 34320186 PMC8476166

[evae169-B33] Naish M, Alonge M, Wlodzimierz P, Tock AJ, Abramson BW, Schmücker A, Mandáková T, Jamge B, Lambing C, Kuo P, et al The genetic and epigenetic landscape of the *Arabidopsis* centromeres. Science. 2021:374(6569):eabi7489. 10.1126/science.abi7489. 34762468 PMC10164409

[evae169-B34] Ou S, Su W, Liao Y, Chougule K, Agda JRA, Hellinga AJ, Lugo CSB, Elliott TA, Ware D, Peterson T, et al Benchmarking transposable element annotation methods for creation of a streamlined, comprehensive pipeline. Genome Biol. 2019:20(1):275. 10.1186/s13059-019-1905-y. 31843001 PMC6913007

[evae169-B35] Pearson WR . An introduction to sequence similarity (“homology”) searching. Curr Protoc Bioinformatics. 2013:42:3.1.1–3.1.8. 10.1002/0471250953.bi0301s42. PMC3820096 23749753

[evae169-B36] Pertea M, Pertea GM, Antonescu CM, Chang T-C, Mendell JT, Salzberg SL. StringTie enables improved reconstruction of a transcriptome from RNA-seq reads. Nat Biotechnol. 2015:33(3):290–295. 10.1038/nbt.3122. 25690850 PMC4643835

[evae169-B37] Platt A, Horton M, Huang YS, Li Y, Anastasio AE, Mulyati NW, Ågren J, Bossdorf O, Byers D, Donohue K, et al The scale of population structure in Arabidopsis thaliana. PLoS Genet. 2010:6(2):e1000843. 10.1371/journal.pgen.1000843. 20169178 PMC2820523

[evae169-B38] Provart NJ, Alonso J, Assmann SM, Bergmann D, Brady SM, Brkljacic J, Browse J, Chapple C, Colot V, Cutler S, et al 50 years of Arabidopsis research: highlights and future directions. New Phytol. 2016:209(3):921–944. 10.1111/nph.13687. 26465351

[evae169-B39] Ranallo-Benavidez TR, Jaron KS, Schatz MC. GenomeScope 2.0 and Smudgeplot for reference-free profiling of polyploid genomes. Nat Commun. 2020:11(1):1432. 10.1038/s41467-020-14998-3. 32188846 PMC7080791

[evae169-B40] Raza A, Hafeez MB, Zahra N, Shaukat K, Umbreen S, Tabassum J, Charagh S, Khan RSA, Hasanuzzaman M. The plant family Brassicaceae: introduction, biology, and importance. In: The plant family Brassicaceae. Singapore: Springer Singapore; 2020. p. 1–43. 10.1007/978-981-15-6345-4_1.

[evae169-B41] Rhie A, Walenz BP, Koren S, Phillippy AM. Merqury: reference-free quality, completeness, and phasing assessment for genome assemblies. Genome Biol. 2020:21(1):245. 10.1186/s13059-020-02134-9. 32928274 PMC7488777

[evae169-B42] Shumate A, Salzberg SL. Liftoff: accurate mapping of gene annotations. Bioinformatics. 2021:37(12):1639–1643. 10.1093/bioinformatics/btaa1016. 33320174 PMC8289374

[evae169-B43] Slater GSC, Birney E. Automated generation of heuristics for biological sequence comparison. BMC Bioinformatics. 2005:6:31. 10.1186/1471-2105-6-31. 15713233 PMC553969

[evae169-B44] Stanke M, Keller O, Gunduz I, Hayes A, Waack S, Morgenstern B. AUGUSTUS: ab initio prediction of alternative transcripts. Nucleic Acids Res. 2006:34(Web Server):W435–W439. 10.1093/nar/gkl200. 16845043 PMC1538822

[evae169-B45] Ter-Hovhannisyan V, Lomsadze A, Chernoff YO, Borodovsky M. Gene prediction in novel fungal genomes using an ab initio algorithm with unsupervised training. Genome Res. 2008:18(12):1979–1990. 10.1101/gr.081612.108. 18757608 PMC2593577

[evae169-B46] The 1001 Genomes Consortium . 1,135 genomes reveal the global pattern of polymorphism in *Arabidopsis thaliana*. Cell. 2016:166(2):481–491. 10.1016/j.cell.2016.05.063. 27293186 PMC4949382

[evae169-B47] Van de Weyer AL, Monteiro F, Furzer OJ, Nishimura MT, Cevik V, Witek K, Jones JDG, Dangl JL, Weigel D, Bemm F. A species-wide inventory of NLR genes and alleles in *Arabidopsis thaliana*. Cell. 2019:178(5):1260–1272.e14. 10.1016/j.cell.2019.07.038. 31442410 PMC6709784

[evae169-B48] Vaser R, Sović I, Nagarajan N, Šikić M. Fast and accurate de novo genome assembly from long uncorrected reads. Genome Res. 2017:27(5):737–746. 10.1101/gr.214270.116. 28100585 PMC5411768

[evae169-B49] Vurture GW, Sedlazeck FJ, Nattestad M, Underwood CJ, Fang H, Gurtowski J, Schatz MC. GenomeScope: fast reference-free genome profiling from short reads. Bioinformatics. 2017:33(14):2202–2204. 10.1093/bioinformatics/btx153. 28369201 PMC5870704

[evae169-B50] Walker BJ, Abeel T, Shea T, Priest M, Abouelliel A, Sakthikumar S, Cuomo CA, Zeng Q, Wortman J, Young SK, et al Pilon: an integrated tool for comprehensive microbial variant detection and genome assembly improvement. PLoS One. 2014:9(11):e112963. 10.1371/journal.pone.0112963. 25409509 PMC4237348

[evae169-B51] Wlodzimierz P, Rabanal FA, Burns R, Naish M, Primetis E, Scott A, Mandáková T, Gorringe N, Tock AJ, Holland D, et al Cycles of satellite and transposon evolution in *Arabidopsis* centromeres. Nature. 2023:618(7965):557–565. 10.1038/s41586-023-06062-z. 37198485

